# Improvement of Visible‐Light H_2_ Evolution Activity of Pb_2_Ti_2_O_5.4_F_1.2_ Photocatalyst by Coloading of Rh and Pd Cocatalysts

**DOI:** 10.1002/chem.202200875

**Published:** 2022-06-16

**Authors:** Kenta Aihara, Kosaku Kato, Tomoki Uchiyama, Shuhei Yasuda, Toshiyuki Yokoi, Akira Yamakata, Yoshiharu Uchimoto, Kazuhiko Maeda

**Affiliations:** ^1^ Department of Chemistry School of Science Tokyo Institute of Technology 2-12-1-NE-2 Ookayama Meguro-ku Tokyo 152-8550 Japan; ^2^ Graduate School of Engineering Toyota Technological Institute 2-12-1 Hisakata, Tempaku Nagoya 468-8511 Japan; ^3^ Present address: Graduate School of Natural Science and Technology Okayama University 3-1-1 Tsushima-naka, Kita-ku Okayama Japan; ^4^ Graduate School of Human and Environmental Studies Kyoto University Yoshidanihonmatsu-cho, Sakyo-ku Kyoto 606-8501 Japan; ^5^ Nanospace Catalysis Unit Institute of Innovative Research Tokyo Institute of Technology Yokohama 226-8503 Japan

**Keywords:** artificial photosynthesis, heterogeneous photocatalysis, oxyfluoride, solar fuels, water splitting

## Abstract

Pb_2_Ti_2_O_5.4_F_1.2_ modified with various metal cocatalysts was studied as a photocatalyst for visible‐light H_2_ evolution. Although unmodified Pb_2_Ti_2_O_5.4_F_1.2_ showed negligible activity, modification of its surface with Rh led to the best observed promotional effect among the Pb_2_Ti_2_O_5.4_F_1.2_ samples modified with a single metal cocatalyst. The H_2_ evolution activity was further enhanced by coloading with Pd; the Rh−Pd/Pb_2_Ti_2_O_5.4_F_1.2_ photocatalyst showed 3.2 times greater activity than the previously reported Pt/Pb_2_Ti_2_O_5.4_F_1.2_. X‐ray absorption fine‐structure spectroscopy, photoelectrochemical, and transient absorption spectroscopy measurements indicated that the coloaded Rh and Pd species, which were partially alloyed on the Pb_2_Ti_2_O_5.4_F_1.2_ surface, improved the electron‐capturing ability, thereby explaining the high activity of the coloaded Rh−Pd/Pb_2_Ti_2_O_5.4_F_1.2_ catalyst toward H_2_ evolution.

## Graphical abstract

**Visible‐light‐induced H_2_** evolution activity of a Pb_2_Ti_2_O_5.4_F_1.2_ photocatalyst was found to be sensitive to the loaded metal cocatalyst. Although Rh was the most effective single‐metal cocatalyst among the tested metals, the activity of Rh/Pb_2_Ti_2_O_5.4_F_1.2_ was further improved by coloading of Pd because it reinforced the ability of Rh to capture photogenerated electrons from Pb_2_Ti_2_O_5.4_F_1.2_.

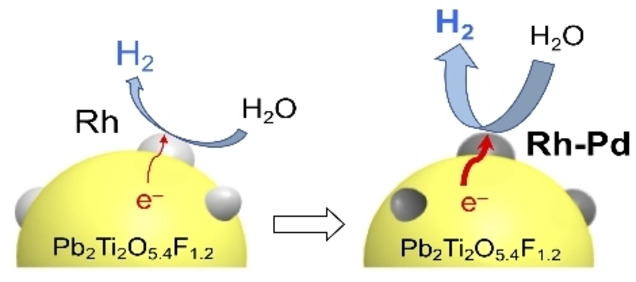

## Introduction

To develop photocatalysts that can absorb visible light, researchers have actively studied mixed‐anion compounds with a controllable bandgap in recent years.[^1^, ^2^, ^3^] Pb_2_Ti_2_O_5.4_F_1.2_ is a visible‐light‐responsive mixed‐anion photocatalyst with a bandgap of 2.4 eV.[^4^, ^5^] The valence‐band maximum (VBM) of Pb_2_Ti_2_O_5.4_F_1.2_ consists of O 2*p* orbitals, whereas that of ordinary mixed‐anion compounds such as oxynitrides and oxysulfides is composed of N 2*p* and S 3*p* orbitals, respectively. Therefore, Pb_2_Ti_2_O_5.4_F_1.2_ is expected to function as a highly stable photocatalyst that does not undergo self‐oxidation by valence‐band holes. However, the activity of the Pb_2_Ti_2_O_5.4_F_1.2_ photocatalyst is unsatisfactory.

**Scheme 1:**
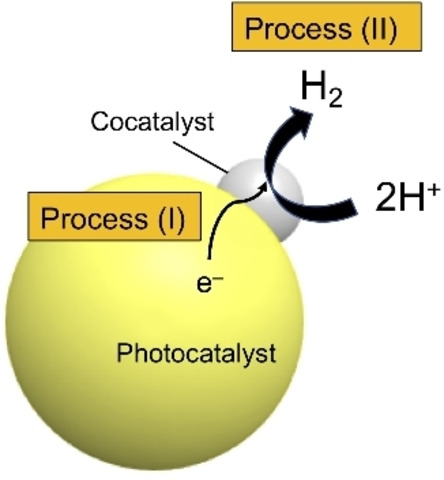
Schematic of electron transfer from a photocatalyst to a loaded cocatalyst (Process I); H_2_ evolution (Process II) is also shown.

Toward efficient H_2_ evolution, cocatalysts, typically in the form of nanoparticles of metals (or metal oxides), are known to strongly affect the photocatalytic activity.[^6^, ^7^, ^8^, ^9^, ^10^, ^11^] A cocatalyst on a semiconductor photocatalyst has two main functions: (1) capturing excited electrons and/or holes and (2) hosting active reaction sites (Scheme 1).[^12^, ^13^] In general, nanoparticulate Pt is a good cocatalyst for H_2_ evolution for many semiconductor photocatalysts because of its excellent catalytic function of proton reduction, as already well documented in the electrochemistry field. However, Pt is not always the best‐performing cocatalyst for a photocatalyst, likely because of the complicated charge‐transfer process at the Pt/photocatalyst interface.[^6^, ^14^]

Therefore, investigating cocatalysts is important for improving the photocatalytic activity of semiconductor photocatalysts, especially those that are newly developed. Thus far, numerous photocatalysts, including metal oxides, sulfides, oxynitrides, and organic polymers, have been investigated. However, to the best of our knowledge, the literature contains no report on the photocatalytic properties of an oxyfluoride for H_2_ evolution with respect to cocatalyst loading. Thus, knowledge of the photocatalytic properties of oxyfluorides remains inadequate.

In the present study, optimal cocatalysts for the Pb_2_Ti_2_O_5.4_F_1.2_ photocatalyst were investigated to improve its H_2_ production activity. In addition, how the optimal cocatalyst works is discussed on the basis of the results of photoelectrochemical and transient absorption spectroscopy measurements.

## Results and Discussion

### Photocatalytic activities of metal‐loaded Pb_2_Ti_2_O_5.4_F_1.2_

**Figure 1:**
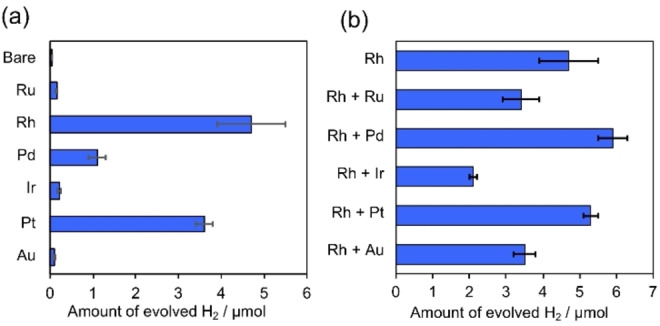
Photocatalytic H_2_ evolution activities of Pb_2_Ti_2_O_5.4_F_1.2_ loaded with (a) various metal cocatalysts (0.5 wt%) and (b) Rh and various metals (0.5 wt% each). Reaction conditions: catalyst, 4 mg (0.5 wt% cocatalyst photodeposited in situ); reaction solution, MeCN:TEOA:H_2_O mixture (89 : 10 : 1 v/v/v) 4 mL; light source, LED lamp (365 nm); reaction time, 20 h.

Various metals (e. g., Ru, Rh, Pd, Ir, Pt, and Au) were investigated as cocatalysts for the Pb_2_Ti_2_O_5.4_F_1.2_ photocatalyst using a batch‐type reactor under 365 nm LED light (see Figure S1a for the spectral irradiance) to identify the optimal metal cocatalyst. These metals are known to function as cocatalysts for photocatalytic H_2_ evolution.[^9^, ^15^] Whereas unmodified Pb_2_Ti_2_O_5.4_F_1.2_ exhibited little activity, Pb_2_Ti_2_O_5.4_F_1.2_ modified with the metals via an in situ photodeposition method demonstrated enhanced activity (Figure 1a). Among the metals examined, Rh resulted in the highest H_2_ generation activity when loaded as a cocatalyst onto Pb_2_Ti_2_O_5.4_F_1.2_.

The H_2_ generation activity was found to be further increased by the coloading of Rh and Pd (Figure 1b). The activity of Rh/Pd‐coloaded Pb_2_Ti_2_O_5.4_F_1.2_ was ∼30 % higher than that of the Rh‐loaded analogue. Coloading of Rh and Pt could moderately improve the H_2_ evolution activity but was not as effective as the coloading of Rh and Pd.

**Figure 2:**
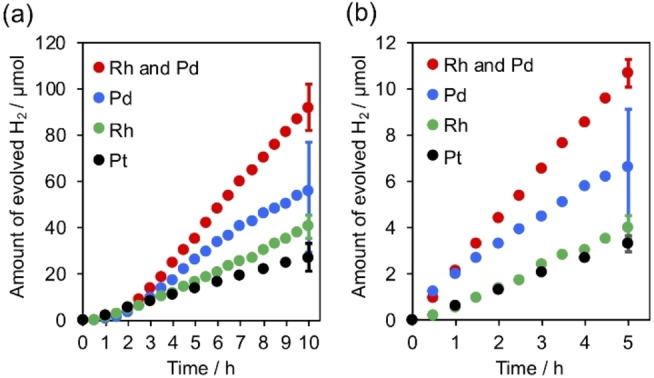
Time courses of H_2_ evolution by modified Pb_2_Ti_2_O_5.4_F_1.2_ photocatalysts under irradiation with (a) UV (*λ* >350 nm) and (b) visible light (*λ* >400 nm). Reaction conditions: catalyst, 100 mg (0.5 wt% cocatalyst photodeposited in situ); reaction solution, MeCN:TEOA mixture (130 : 10 v/v) containing 1 mL water, 140 mL; light source, 300 W Xe lamp.

Figure 2 shows the time courses of H_2_ evolution by the Rh/Pd‐coloaded Pb_2_Ti_2_O_5.4_F_1.2_, along with that of the previously reported Pt‐loaded analogue.^[16]^ These experiments were conducted using a closed gas circulation system with a 300 W Xe lamp, which could provide more photons in visible light region (Figure S1b). Under this condition, the Pd/Pb_2_Ti_2_O_5.4_F_1.2_ showed a relatively high initial H_2_ evolution rate; however, the H_2_ evolution rate degraded over time, with a large experimental error. By contrast, the coloaded photocatalyst produced H_2_ stably and was ∼3.2 times more active than the previously reported Pt‐loaded photocatalyst both under UV irradiation and under visible‐light irradiation. The enhancement factor of 3.2 achieved by the coloaded Rh and Pd under high‐intensity light irradiation (Figure 2b) was greater than that obtained under LED irradiation (Figure 1b). This difference suggests that the activity improvement by coloading is more pronounced under irradiation with high‐intensity light.

### Characterization of the loaded Rh and Pd species

**Figure 3:**
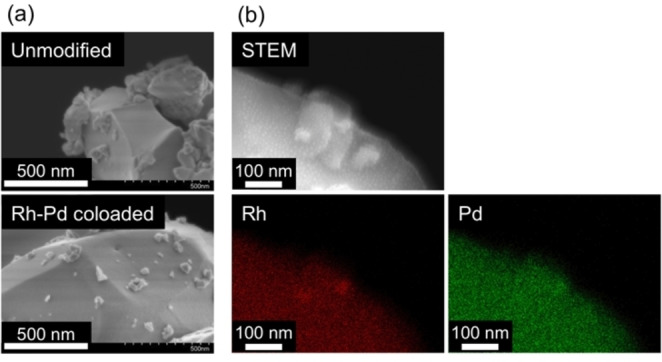
a) SEM and b) STEM/EDS mapping images of the Rh−Pd/Pb_2_Ti_2_O_5.4_F_1.2_.

We attempted to examine how Rh and Pd were deposited onto the Pb_2_Ti_2_O_5.4_F_1.2_. However, obtaining a clear picture was difficult. As shown in Figure 3, scanning electron microscopy (SEM) observations revealed that the surface of the unmodified Pb_2_Ti_2_O_5.4_F_1.2_ was rough, containing numerous irregularly shaped smaller particles. The Rh−Pd/Pb_2_Ti_2_O_5.4_F_1.2_ exhibited a similar morphological character, which made it difficult to distinguish any photodeposited species from the smaller Pb_2_Ti_2_O_5.4_F_1.2_ particles. We conducted energy‐dispersive X‐ray spectroscopy (EDS) measurements with care to avoid beam‐damage to the sample. In some cases, Rh and Pd signals were observed close to each other (Figure 3b); however, we could not determine whether the same was true for other locations. Observations by transmission electron microscopy (TEM) also failed, as the electron densities of Rh and Pd are very similar and the sample could not withstand the strong electron beams during the TEM observations.

**Figure 4:**
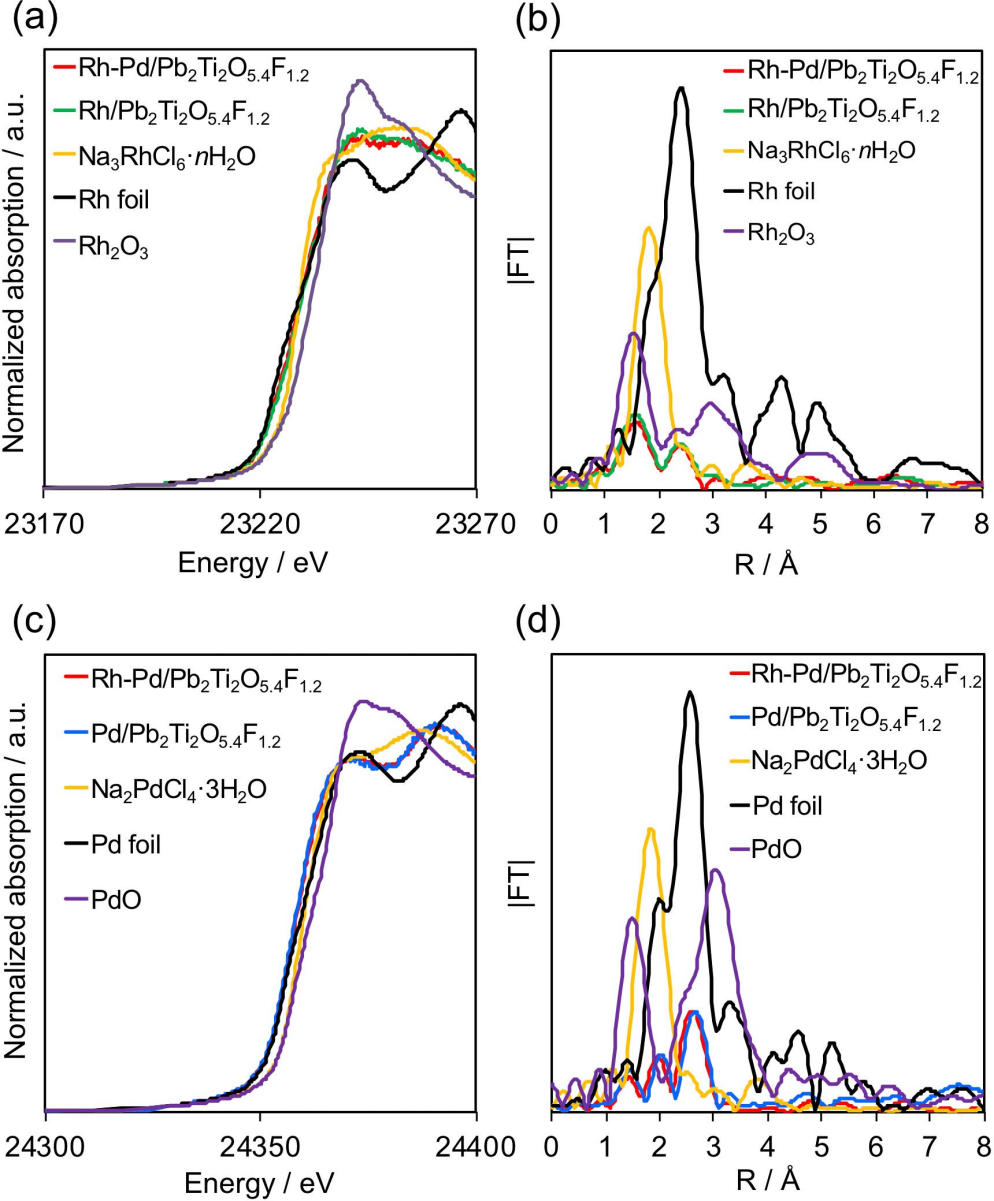
Results of XAFS measurement for modified Pb_2_Ti_2_O_5.4_F_1.2_. Rh−K edge (a) XANES and (b) FT‐EXAFS spectra. Pd−K edge (c) XANES and (d) FT‐EXAFS spectra. Note that FT‐EXAFS cannot distinguish Pd−Rh, Pd−Pd, and Rh−Rh bonds because Pd and Rh have similar atomic numbers (i. e., approximately the same backscattering amplitude).

To examine the electronic state, the valence state of Rh and Pd species photodeposited onto Pb_2_Ti_2_O_5.4_F_1.2_ were characterized by XAFS measurement. The Rh−K edge X‐ray absorption near‐edge structure (XANES) spectra for Rh/Pb_2_Ti_2_O_5.4_F_1.2_, Rh−Pd/Pb_2_Ti_2_O_5.4_F_1.2_, and some reference materials are shown in Figure 4a. The Rh−K edge XANES spectrum for Rh−Pd/Pb_2_Ti_2_O_5.4_F_1.2_ is similar to that for Rh/Pb_2_Ti_2_O_5.4_F_1.2_. By comparing the spectra of the samples with those of the reference materials, we found that the loaded Rh species in the two samples was a mixture of oxide and metal. The Rh−K edge XANES spectrum for the Rh−Pd/Pb_2_Ti_2_O_5.4_F_1.2_ could be reproduced by a linear combination of the spectra of the Rh foil and Rh_2_O_3_ references, and the Rh:Rh_2_O_3_ ratio for the Rh−Pd/Pb_2_Ti_2_O_5.4_F_1.2_ sample was found to be 56 : 44. However, the Rh:Rh_2_O_3_ ratio for the Rh/Pb_2_Ti_2_O_5.4_F_1.2_ was 52 : 48. Therefore, the Rh−Pd/Pb_2_Ti_2_O_5.4_F_1.2_ sample contained a higher density of metallic Rh than the Rh/Pb_2_Ti_2_O_5.4_F_1.2_ sample.

Fourier transforms (FTs) of the *k* ^2^‐weighted Rh−K edge EXAFS data confirmed the existence of Rh−O and metallic Rh‐*M* (*M*=Rh or Pd) bonds at *R* ≈1.5 and 2.4 Å, respectively. However, no peak derived from Rh‐(O)‐Rh bonding (e. g., at ∼3 Å) was observed, suggesting the absence of bulk Rh_2_O_3_ species. The absence of the Rh−Cl peak for Na_3_RhCl_6_ ⋅ *n*H_2_O also indicates complete decomposition of the precursor during the photodeposition process.

As shown in Figure 4c, the peaks in the Pd−K edge XANES spectra for the two samples were located at slightly lower energies than those for the Pd foil reference, indicating that the Pd species are more electron‐rich than Pd foil. This result might be attributable to an electron‐donation effect of the Pb_2_Ti_2_O_5.4_F_1.2_ support, which is an n‐type semiconductor.[^4^, ^17^]

Although no difference was observed in the Pd−K edge XANES spectra between singly loaded and coloaded samples, the FT‐EXAFS spectra indicated somewhat different bonding natures in these samples (Figure 4d). The Pd foil gave a strong peak at ∼2.7 Å, which is attributed to Pd−Pd bonds. However, the peaks due to Pd‐*M* bonds (*M*=Pd or Rh) in the spectra of the Pd/Pb_2_Ti_2_O_5.4_F_1.2_ and Rh−Pd/Pb_2_Ti_2_O_5.4_F_1.2_ samples were located at longer distances than the Pd−Pd peak position in the spectrum of the Pd foil reference. This result indicates that the bond length for Pd‐*M* in the two samples was larger than that for Pd−Pd in the Pd foil. This finding is consistent with the results of the Pd−K edge XANES measurements (Figure 4c), which indicated that the Pd species in the two samples was in an electron‐rich state. The Pd‐*M* bond length in the Rh−Pd/Pb_2_Ti_2_O_5.4_F_1.2_ was smaller than that in the Pd/Pb_2_Ti_2_O_5.4_F_1.2_, strongly suggesting that partial alloying occurred between Rh and Pd in the Rh−Pd/Pb_2_Ti_2_O_5.4_F_1.2_ given that the lattice constants of Rh and Pd are *a*=3.71559 and 3.8898 Å, respectively. Rh and Pd have been reported to form an alloy over the full composition range.[^18^, ^19^, ^20^] However, the formation of a core/shell‐like structure (i. e., Rh on Pd or Pd on Rh) is unlikely given the surface atomic ratios of Rh/Ti and Pd/Ti, which were almost constant between singly and doubly loaded samples (Table S1).

The valence states of Rh and Pd species were also investigated by X‐ray photoelectron spectroscopy (XPS). The XPS measurements lead to the same conclusions as the XAFS measurements (Figure S2). Although the relative locations of the Rh and Pd species in the Rh−Pd/Pb_2_Ti_2_O_5.4_F_1.2_ could not be identified by electron microscopy, XAFS measurements gave clearer information about their “locations” based on their electronic states.

### Factors affecting the photocatalytic activity

To investigate whether the reduction or oxidation reaction was promoted by the coloading of Rh and Pd, we conducted photoelectrochemical measurements using modified Pb_2_Ti_2_O_5.4_F_1.2_ electrodes under visible light. Because an n‐type semiconductor shows a rectifying action, its photooxidation ability can be monitored using photocurrent measurements.[^21^, ^22^]

**Figure 5:**
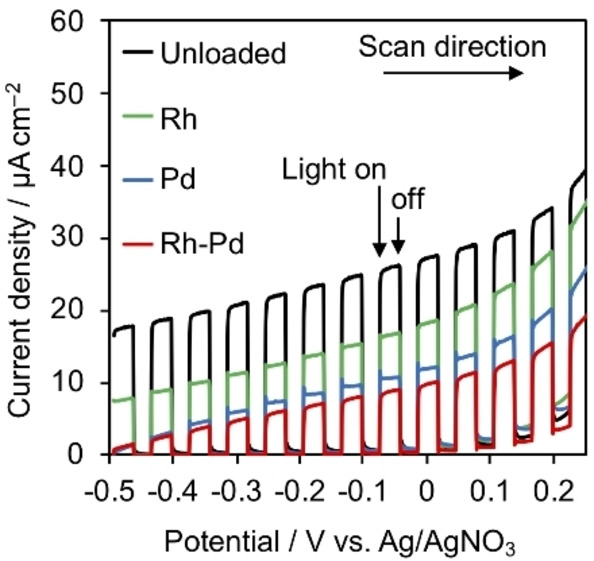
Current‐voltage curves obtained from modified Pb_2_Ti_2_O_5.4_F_1.2_ electrodes under intermittent visible light (*λ*>400 nm) in acetonitrile containing dissolved Et_4_NBF_4_, TEOA, and H_2_O. Scan rate: 10 mV s^−1^. Light source: 300 W Xe lamp. Irradiation area: 5.3 cm^2^. [Ag/AgNO_3_ at pH 7]=[NHE at pH 7] – 0.56.

Figure 5 shows current‐voltage curves for modified Pb_2_Ti_2_O_5.4_F_1.2_ electrodes under intermittent irradiation with visible light (*λ* >400 nm) in the presence of triethanolamine (TEOA) as an electron donor. These results show that the photocurrent density for TEOA oxidation decreased in the order Pb_2_Ti_2_O_5.4_F_1.2_, Rh/Pb_2_Ti_2_O_5.4_F_1.2_, Pd/Pb_2_Ti_2_O_5.4_F_1.2_, and Rh−Pd/Pb_2_Ti_2_O_5.4_F_1.2_. Thus, the loading of Rh or Pd as well as their coloading lowered the efficiency of photooxidation reactions on the Pb_2_Ti_2_O_5.4_F_1.2_ surface, most likely because the loaded metal species reduced the available area for oxidation reactions. Nevertheless, the photoreduction activity of the Pb_2_Ti_2_O_5.4_F_1.2_ during H_2_ evolution was improved by Rh and Pd loading (Figure 2). This result suggests that the promotional effect of Rh and Pd coloading on the Pb_2_Ti_2_O_5.4_F_1.2_ resulted from improvements of the photoreduction processes.

**Figure 6:**
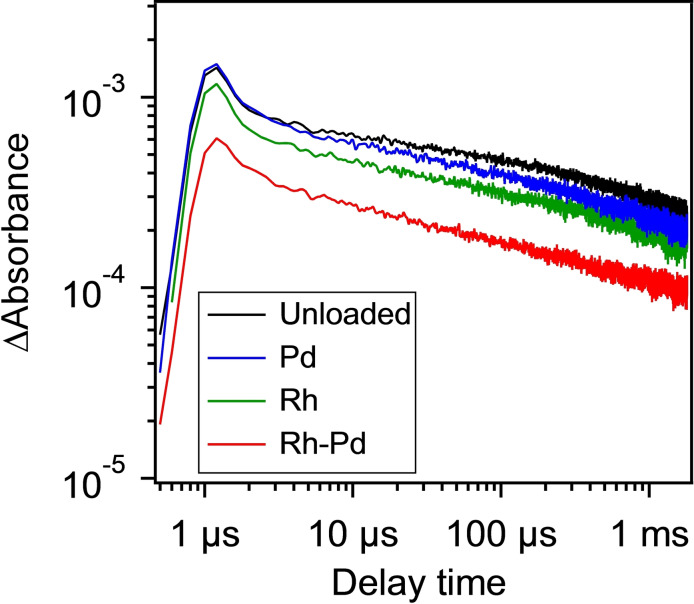
Transient absorption intensity decay curves at 2000 cm^−1^ for modified Pb_2_Ti_2_O_5.4_F_1.2_ samples.

To further investigate the behavior of photogenerated electrons in the modified Pb_2_Ti_2_O_5.4_F_1.2_, we carried out transient absorption measurements. Figure 6 shows the decay kinetics of the transient absorption signals recorded at 2000 cm^−1^, which are attributed to photogenerated free electrons and/or shallowly trapped electrons in the Pb_2_Ti_2_O_5.4_F_1.2_.[^23^, ^24^] A smaller Δabsorbance and its faster decay indicate more efficient trapping of photogenerated electrons.[^23^, ^24^] The Rh/Pb_2_Ti_2_O_5.4_F_1.2_ showed a smaller Δabsorbance than Pd/Pb_2_Ti_2_O_5.4_F_1.2_ and bare Pb_2_Ti_2_O_5.4_F_1.2_, indicating that Rh exhibited better electron‐capturing ability than Pd on the Pb_2_Ti_2_O_5.4_F_1.2_. More importantly, Rh−Pd/Pb_2_Ti_2_O_5.4_F_1.2_ exhibited much smaller Δabsorbance than Rh/Pb_2_Ti_2_O_5.4_F_1.2_, indicating that the electron‐capturing property of Rh could be improved by Pd coloading and explaining the enhanced photocatalytic activity for H_2_ evolution.

However, it is noted that the difference in H_2_ evolution activity between the Rh/Pb_2_Ti_2_O_5.4_F_1.2_ and Pd/Pb_2_Ti_2_O_5.4_F_1.2_ under high light intensity conditions cannot simply be explained in terms of the electron‐capturing ability of metal cocatalyst. The behavior of the Pd/Pb_2_Ti_2_O_5.4_F_1.2_ is somewhat strange, in that it showed relatively large fluctuation in the H_2_ yield compared to the Rh/Pb_2_Ti_2_O_5.4_F_1.2_ and Rh−Pd/Pb_2_Ti_2_O_5.4_F_1.2_ under high light intensity conditions (Figure 2). This suggests that there are factors that are currently beyond our control regarding the preparation of Pd/Pb_2_Ti_2_O_5.4_F_1.2_ and its photocatalytic properties. At present, we could not fully elucidate photocatalytic behavior of the Pd/Pb_2_Ti_2_O_5.4_F_1.2_, which requires more systematic investigations such as the light intensity effect (although beyond the scope of the present work).

## Conclusion

The Pb_2_Ti_2_O_5.4_F_1.2_ photocatalyst exhibited improved visible‐light‐induced H_2_ evolution activity when loaded with various metal cocatalysts. Among the examined cocatalysts, Rh exhibited the best performance, and coloading with Pd further improved the activity. Photoelectrochemical and transient absorption spectroscopy measurements revealed that these cocatalysts effectively accelerate the photoreduction processes. In particular, the coloading of Pd improved the electron‐capturing ability of Rh.

However, apparent quantum yield of the Rh−Pd/Pb_2_Ti_2_O_5.4_F_1.2_ photocatalyst was at most 0.25 % at 420 nm even after optimization of the loading amount of Rh and Pd (see Figure S3 for the detail). Therefore, further development of the method for cocatalyst loading as well as for synthesis of Pb_2_Ti_2_O_5.4_F_1.2_ photocatalyst is required. It is also noted that at present, how the H_2_ evolution occurs on the Rh−Pd cocatalyst remains unknown. A theoretical approach^[25]^ may elucidate the reaction mechanism.

## Experimental Section

**Materials and reagents**: PbO (99.9 %) and TiO_2_ (rutile, 99.99 %) were purchased from Rare Metallic. PbF_2_ (99.9 %) was purchased from Mitsuwa Pure Chemicals. NaCl (>99.5 %), acetonitrile (MeCN; >99.5 %), and TEOA (>98.0 %) were purchased from Kanto Chemical Co. CsCl (>99.9 %) was purchased from Wako Pure Chemicals. For the loading of metal cocatalysts, Ni(NO_3_)_2_ ⋅ 6H_2_O (>99.5 %, Kanto Chemical), RuCl_3_ ⋅ 3H_2_O (>36 % as Ru, Wako Pure Chemical Industries), Na_3_RhCl_6_ ⋅ *n*H_2_O (17.4 % as Rh, Mitsuwa Pure Chemicals), Na_2_PdCl_4_ ⋅ 3H_2_O (>95.0 %, Wako Pure Chemical Industries), Na_2_IrCl_6_ ⋅ 6H_2_O (>34.0 % as Ir, Wako Pure Chemical Industries), H_2_PtCl_6_ ⋅ 6H_2_O (98.5 %, Kanto Chemical), and HAuCl_4_ ⋅ 4H_2_O (99 %, Kanto Chemical) were used. All chemicals were used without further purification.

**Synthesis of Pb_2_Ti_2_O_5.4_F_1.2_ and alkaline chloride treatment**: The Pb_2_Ti_2_O_5.4_F_1.2_ photocatalyst was synthesized by a solid‐state method.^[26]^ PbO, TiO_2_, and PbF_2_ were mixed in a molar ratio of 1.4 : 2:0.6 and ground under methanol. The mixture was pelletized and then heated in an evacuated Pyrex tube with Pt foil at 823 K for 12 h (ramp rate of 10 K min^−1^).

The as‐synthesized Pb_2_Ti_2_O_5.4_F_1.2_ (250 mg), NaCl, and CsCl were mixed in a Pb_2_Ti_2_O_5.4_F_1.2_ : NaCl : CsCl molar ratio of 1 : 1.4 : 2.6 and then ground. The mixture was subsequently sealed in an evacuated Pyrex tube, which was heated at 723 K for 5 h (ramp rate of 1 K min^−1^). After the mixture naturally cooled to room temperature, the product was separated from the residual alkaline chloride by washing with distilled water several times, and was finally dried in an oven at 343 K. This NaCl‐CsCl treatment has been reported to improve the photocatalytic activity of Pb_2_Ti_2_O_5.4_F_1.2_ for H_2_ evolution, because of alkaline titanate species formed by the treatment on the Pb_2_Ti_2_O_5.4_F_1.2_ that offer favorable reaction sites for proton reduction.^[16]^ In the present work, the NaCl‐CsCl‐treated Pb_2_Ti_2_O_5.4_F_1.2_ was used as a photocatalyst.

**Characterization**: A scanning electron microscope (SU9000 (Hitachi)) equipped with an EDS apparatus (EDAX, TEAM™ EDS system) was used to investigate the morphology and elemental distribution of the materials. XPS was conducted using an ESCA‐3400 X‐ray photoelectron spectrometer (Shimadzu). The binding energies were calibrated by referencing the C 1s peak (285.0 eV) for each sample.

Rh and Pd K‐edge XAFS measurements were conducted on the BL‐14B2 beamline of SPring‐8 (Proposal No. 2020A1895). The X‐ray absorption spectra were recorded in fluorescence mode at room temperature using a Si(311) two‐crystal monochromator. A pair of Rh‐coated mirrors were used to eliminate higher harmonics. The XANES spectra were analyzed using the Athena software package.^[27]^

Transient absorption measurements were conducted using a purpose‐built spectrometer described previously.^[23]^ The experimental details are given elsewhere.^[28]^ Briefly, photocatalyst samples coated onto a CaF_2_ plate at a density of 1.5 mg cm^−2^ were photoexcited under N_2_ (20 Torr) using 420 nm pulses from a Nd:YAG laser (Continuum Surelite; duration, 6 ns; power, 5 mJ; repetition rate, 1 Hz).

**Photocatalytic reactions**: Photocatalytic reactions were conducted in a merry‐go‐round‐type reaction apparatus using an LED (365 nm) light source, Iris‐MG (CELL System), for the purpose of first screening metal cocatalysts. Metal cocatalysts were loaded on the surface of Pb_2_Ti_2_O_5.4_F_1.2_ by an in situ photodeposition method.^[29]^ In each trial, 4 mg of Pb_2_Ti_2_O_5.4_F_1.2_ was dispersed in a test‐tube reaction cell (11 mL capacity) containing 4 mL of reaction solution (MeCN:TEOA:H_2_O=89 : 10 : 1, v/v/v). The use of MeCN as the main solvent is to ensure good dispersion of the hydrophobic Pb_2_Ti_2_O_5.4_F_1.2_ particles.^[4]^ The precursor of the metal was dissolved in the reaction solution at a ratio of 0.5 wt% with respect to the Pb_2_Ti_2_O_5.4_F_1.2_ unless otherwise stated. After the solution was purged with Ar gas, it was irradiated with LED light for 20 h. The generated gases were analyzed using a gas chromatograph equipped with a thermal conductivity detector (GL Science GC323).

Photocatalytic reactions were also conducted using a Pyrex top‐irradiation reaction vessel connected to a closed gas circulation system. In each trial, 100 mg of the photocatalyst was suspended in 140 mL of MeCN‐TEOA mixed solution (13 : 1 v/v) containing 1 mL of water and a precursor of a metal source. After the solution was outgassed, it was irradiated with UV light (*λ*>350 nm) for 10 h using a 300 W Xe lamp (Cermax, PE300BF) fitted with a cold mirror (CM‐1), where an output current of 20 A was applied. After the 10 h UV irradiation and subsequent removal of gaseous products, the photocatalyst suspension was irradiated with visible light (*λ*>400 nm) passed through a cutoff filter (L42). The evolved gases were analyzed by gas chromatography (GC‐8 A equipped with a thermal conductivity detector and an MS‐5 A column (Shimadzu)); Ar was used as the carrier gas. Apparent quantum yield was measured in the same manner as reported previously.^[16]^

**Photoelectrochemical measurements**: Photoelectrochemical measurements were carried out with a potentiostat (HZ‐pro, Hokuto Denko) and an electrochemical cell at room temperature. The cell was made of Pyrex glass and was a three‐electrode‐type system with a Pt wire and a Ag/AgNO_3_ electrode as the counter and reference electrodes, respectively. The working electrode, Pb_2_Ti_2_O_5.4_F_1.2_/FTO, was prepared by an electrophoretic deposition method according to our previous method,^[16]^ with some modifications (time of deposition, 2.5 min; without TiCl_4_ treatment). A mixed solution of MeCN‐TEOA‐H_2_O (130 : 10 : 1 v/v/v) dissolved with 0.1 M tetraethylammonium tetrafluoroborate (Et_4_NBF_4_; 99 %, Sigma‐Aldrich) was used as the electrolyte solution and was saturated with Ar gas prior to the electrochemical measurements. The light source was a 300 W Xe lamp (Cermax, PE300BF) equipped with a cutoff filter (L42) to emit visible light. The irradiation area was ∼5.3 cm^2^.

## Conflict of interest

The authors declare no conflict of interest.

1

## Supporting information

As a service to our authors and readers, this journal provides supporting information supplied by the authors. Such materials are peer reviewed and may be re‐organized for online delivery, but are not copy‐edited or typeset. Technical support issues arising from supporting information (other than missing files) should be addressed to the authors.

Supporting Information

## Data Availability

The data that support the findings of this study are available from the corresponding author upon reasonable request.
