# Supplementary material for: Improvement of Visible‐Light H2 Evolution Activity of Pb2Ti2O5.4F1.2 Photocatalyst by Coloading of Rh and Pd Cocatalysts
Source: Chemistry. 2022 Jun 16;28(43):e202200875. doi: 10.1002/chem.202200875 (PMC9401856; doi:10.1002/chem.202200875)
Supplement: Supplementary file 1 — Supporting Information [file CHEM-28-0-s001.pdf]

Chemistry–A European Journal

Supporting Information

Improvement of Visible-Light H_2 Evolution Activity of $Pb_2Ti_2O_{5.4}F_{1.2}$ Photocatalyst by Coloading of Rh and Pd Cocatalysts

Kenta Aihara, Kosaku Kato, Tomoki Uchiyama, Shuhei Yasuda, Toshiyuki Yokoi, Akira Yamakata, Yoshiharu Uchimoto, and Kazuhiko Maeda*

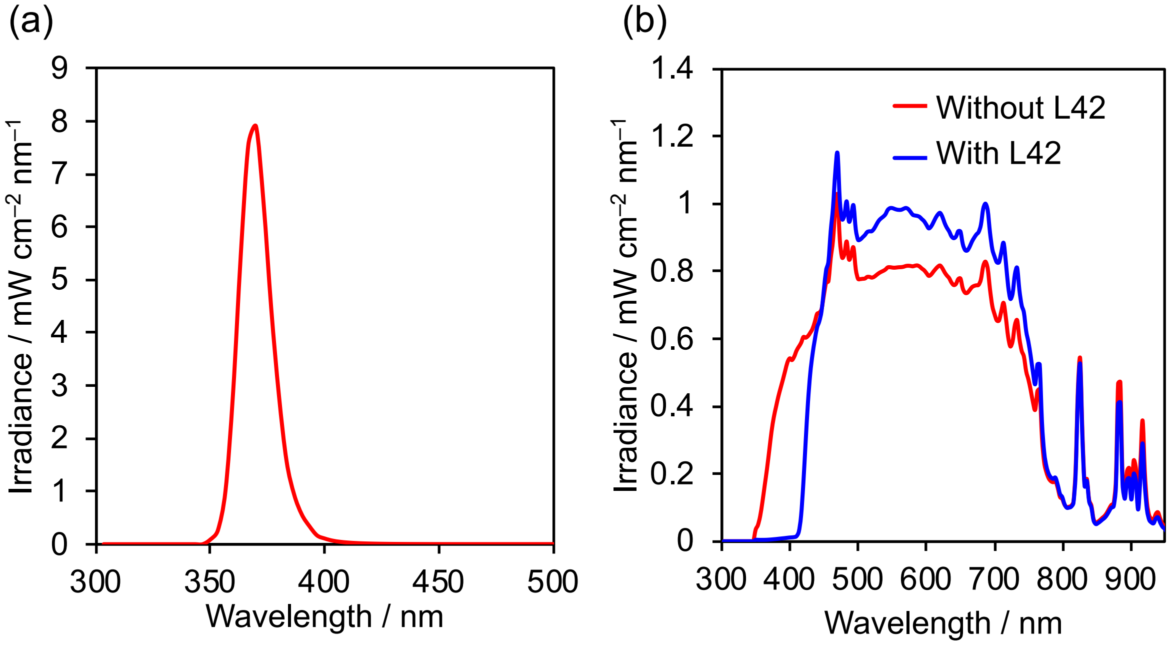

Figure S1. Spectral irradiance of (a) the LED and (b) the 300 W xenon lamps (with and without a L42 cutoff filter). The spectral irradiance of the LED and the 300 W Xe lamp was measured by a spectroradiometer (Eko Instruments, LS-100). The intensities of the incident light from the 300 W Xe lamp hitting the reaction cell were 110 and 74 mW cm^{-2} at the wavelength range of 350–510 nm and 400–510 nm (with the L42 filter), respectively. Note that it was not possible to precisely measure the total light intensity from the LED due to a technical issue. The spectral irradiance shown in the panel a was recorded at the closest position to the LED lamp.

Additional discussion the difference in the H_2 evolution rates between Figures 1 and 2

The much lower H_2 evolution rates in Figure 1 compared to those in Figure 2 can be explained in terms of the incident photon numbers, rather than the incident photon wavelength. That is, we think that the incident photon numbers in the experiment shown in Figure 1 were much lower than those in Figure 2.

This idea is supported by our previous work on Pt-loaded $Pb_2Ti_2O_{5.4}F_{1.2}$ (*J. Mater. Chem. A* **2020**, *8*, 9099–9108), which showed that the apparent quantum yields for H_2 evolution were 0.83 and 0.26% at 365 and 420 nm, respectively, under the identical light intensity conditions (15–18 mW). Therefore, we should consider that there is ~3 times difference in the absolute photocatalytic activity of the $Pb_2Ti_2O_{5.4}F_{1.2}$ (i.e., apparent quantum yield) between the two wavelength conditions.

Obviously, the H_2 evolution rates reported in Figure 1 were an order of magnitude lower than those in Figure 2. Therefore, the difference in the H_2 evolution rate between Figures 1 and 2 is considered to be mainly due to the difference in the number of incident photons from different light sources with different intensities.

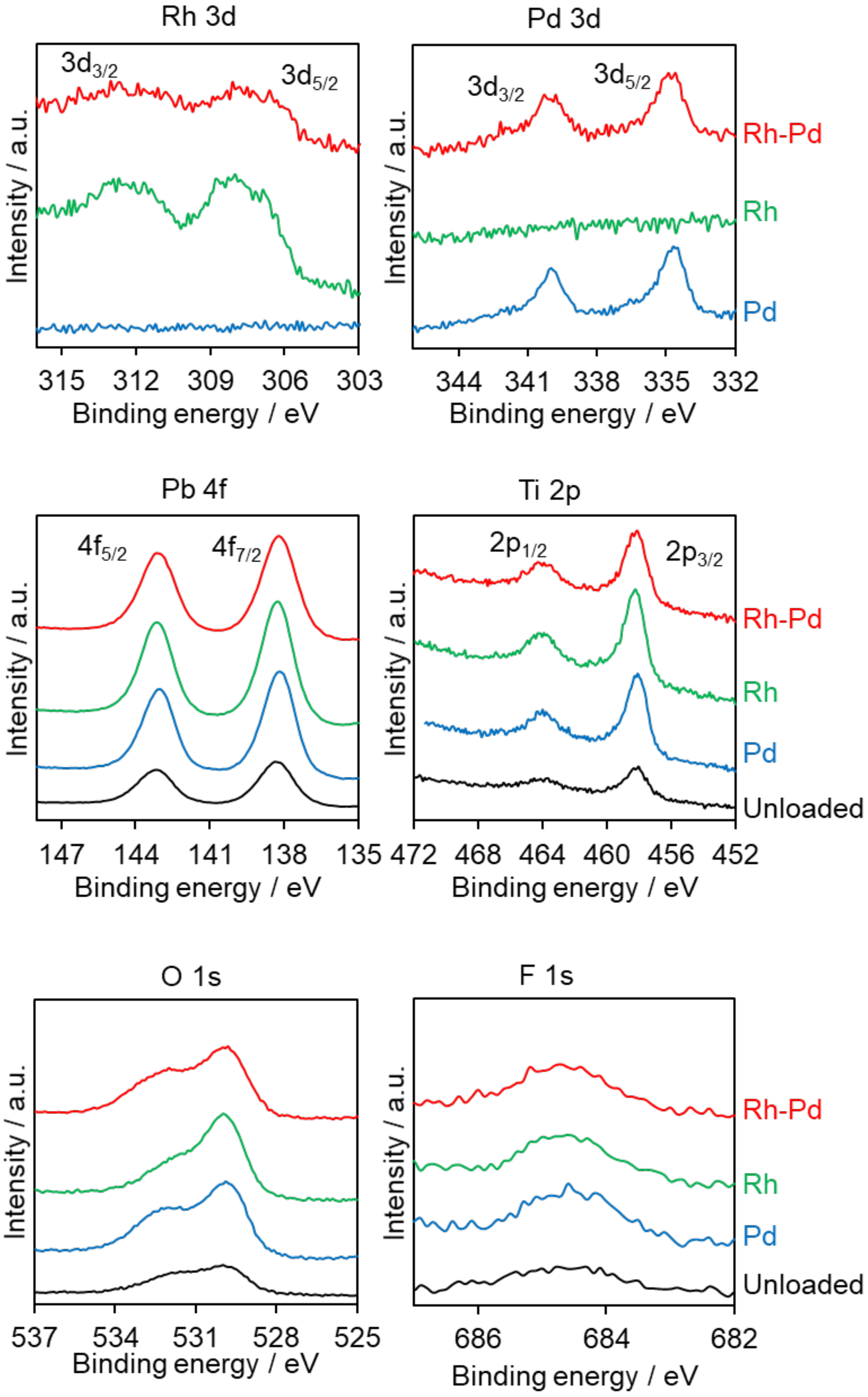

Figure S2. Rh 3d, Pd 3d, Pb 4f, Ti 2p, O 1s, and F 1s XPS spectra for modified $Pb_2Ti_2O_{5.4}F_{1.2}$.

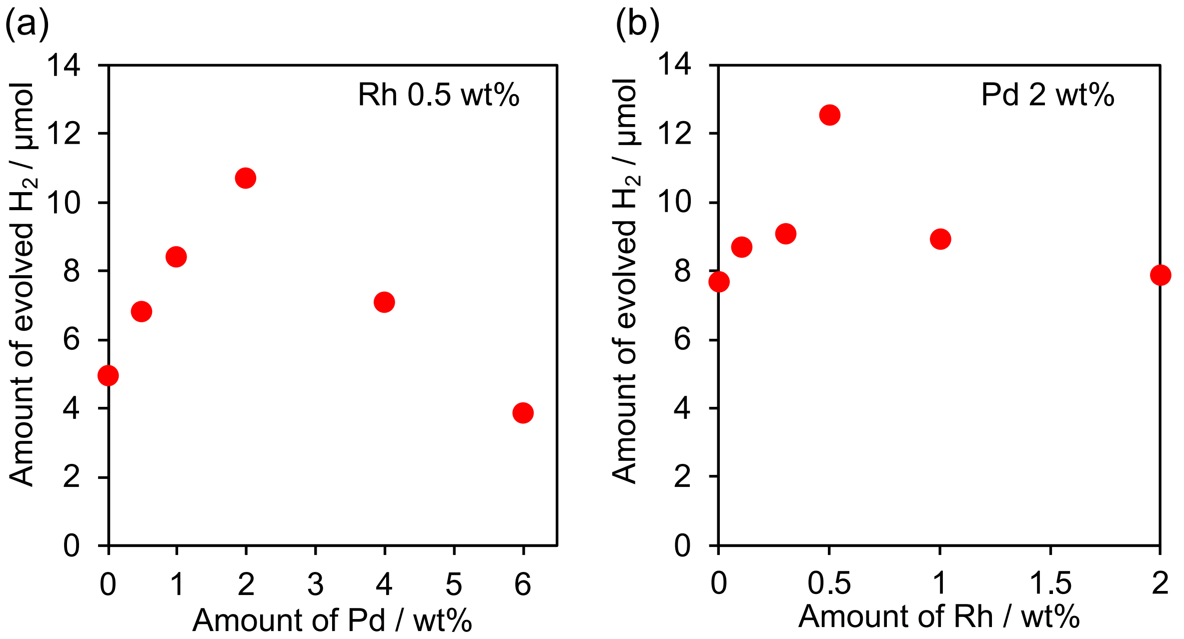

Figure S3. Photocatalytic H_2 evolution activities of $Pb_2Ti_2O_{5.4}F_{1.2}$ loaded with (a) different amounts of Pd and 0.5 wt% Rh, and (b) different amounts of Rh and 2 wt% Pd. Reaction conditions: catalyst, 4 mg (cocatalyst photodeposited in situ); reaction solution, MeCN:TEOA:H_2O mixture (89:10:1 v/v/v) 4 mL; light source, LED lamp (365 nm); reaction time, 20 h.

Table S1. Surface atomic ratios for modified $Pb_2Ti_2O_{5.4}F_{1.2}$, which were estimated from each XPS peak area

Loaded cocatalyst	Pd/Ti	Rh/Ti	F/Ti	Pb/Ti
Rh-Pd	0.04	0.06	0.38	1.65
Rh	n.d.	0.07	0.27	1.43
Pd	0.05	n.d.	0.33	1.51
